# Eleven percent intact PGM3 in a severely immunodeficient patient with a novel splice-site mutation, a case report

**DOI:** 10.1186/s12887-018-1258-9

**Published:** 2018-08-29

**Authors:** Karin E. Lundin, Qing Wang, Abdulrahman Hamasy, Per Marits, Mehmet Uzunel, Valtteri Wirta, Ann-Charlotte Wikström, Anders Fasth, Olov Ekwall, C.I. Edvard Smith

**Affiliations:** 1 Clinical Research Center, Novum, Department of Laboratory Medicine, Karolinska Institutet, SE-141 86 Stockholm, Sweden; 2 Present address: Department of Clinical Analysis, College of Pharmacy, Hawler Medical University, Erbil, Kurdistan Region Iraq; 3 Department of Clinical Immunology, Karolinska University Hospital, Huddinge, SE-14186 Stockholm, Sweden; 4 Science for Life Laboratory, Department of Microbiology, Tumor and Cell Biology, Karolinska Institutet, SE-171 65 Stockholm, Sweden; 5 Science for Life Laboratory, School of Biotechnology, KTH Royal Institute of Technology, SE-171 65 Stockholm, Sweden; 6 Department of Pediatrics, Institute of Clinical Sciences, Sahlgrenska Academy at University of Gothenburg, SE-416 85 Gothenburg, Sweden; 7 Department of Rheumatology and Inflammation Research, Institute of Medicine, Sahlgrenska Academy at University of Gothenburg, SE-416 85 Gothenburg, Sweden

**Keywords:** Congenital disorder of glycosylation, Hyper-IgE, N-acetylglucosamine-phosphate mutase, PGM3 enzyme activity, Phosphoglucomutase 3, Primary immunodeficiency, Splice-modifying mutation

## Abstract

**Background:**

A novel immunodeficiency, frequently accompanied by high serum-IgE, and caused by mutations in the *PGM3* gene was described in 2014. To date there are no unique phenotype characteristics for PGM3 deficiency. *PGM3* encodes a carbohydrate-modifying enzyme, phosphoglucomutase 3. Null-mutations are quite likely lethal, and to date only missense mutations or small deletions have been reported. Such mutations frequently cause a combination of reduced enzyme activity and protein instability, complicating determination of the enzyme level needed for survival. Here we present the first patient with a homozygous splice-modifying mutation in the *PGM3* gene. An A > G substitution at position c.871 + 3 (transcript NM_001199917) is causing a deletion of exon 7 in the majority of *PGM3* transcripts. In addition, this case further increases the clinical phenotypes of immunodeficiency caused by *PGM3* mutations.

**Case presentation:**

We describe the symptoms of a 3-year-old girl who was severely growth retarded, had vascular malformations, extensive eczema, multiple food-allergies, and was prone to infections. Unlike the majority of reported PGM3 deficient patients she lacked skeletal dysplasia and had normal neurocognitive development. In addition to the high serum-IgE, she displayed altered T cell numbers with reduced naïve CD4^+^ and CD8^+^ T-cells, increased number of activated effector memory CD8^+^ T cells and aberrant T-cell functions.

The patient was homozygous for a new hypomorphic, splice-modifying mutation in the *PGM3* gene, causing severely reduced mRNA levels. In the patient’s cells, we observed 5% intact mRNA and approximately 11% of the protein levels seen in healthy controls.

Treatment with allogeneic hematopoietic stem cell therapy was planned, but unfortunately the clinical condition deteriorated with multi-organ failure, which led to her death at 3 years of age.

**Conclusions:**

There is still no specific phenotype identified that distinguishes immunodeficiency caused by *PGM3* mutations from other forms of immunodeficiency. The patient described here yields new information on the phenotypic variability among these patients. In addition, since all the synthesized protein is wild-type, it is possible for the first time to estimate the enzyme activity in vivo. The results suggest that1/10 of the normal PGM3 level is sufficient for survival but that it is insufficient for accurate carbohydrate processing.

**Electronic supplementary material:**

The online version of this article (10.1186/s12887-018-1258-9) contains supplementary material, which is available to authorized users.

## Background

The number of identified genes in which mutations have been shown to induce immunodeficiency has increased considerably as a result of the use of next generation sequencing techniques and the possibility of carrying out whole exome and whole genome analysis [1–3]. A newly identified gene causing primary immunodeficiency is *PGM3* that encodes phosphoglucomutase 3 (PGM3) [4, 5]. Although there are still no unique phenotype characteristics associated with mutations in the *PGM3* gene, the majority, but not all patients with *PGM3* mutations, present with a pronounced high serum IgE, in combination with recurrent staphylococcal skin abscesses, sinopulmonary infections, and severe eczema [4–8]. Recently two patients with severe immunodeficiency but without eczema were also described [9]. Other genes causing symptoms of Hyper-IgE syndrome when mutated are *STAT3*, which is the predominating cause, *DOCK8* and *TYK2*, reviewed in [10, 11].

PGM3 is a key enzyme in the uridine diphosphate N-acetylglucosamine (UDP-GlcNac) synthesis pathway, in which it converts GlcNAc-6-phosphate to GlcNAc-1-phosphate. UDP-GlcNac, is an important building block for both N- and O-linked glycosylation. Congenital disorders in glycosylation can influence many cellular functions, induce developmental changes such as skeletal and mental disturbance and cause severe immunological defects [10, 12]. In mice, it was found that a null mutation in the *Pgm3* gene is lethal [13]. Furthermore, until recently all reported studied patients presenting with *PGM3* mutations carry homozygous or compound heterozygous missense mutations [4–9], or a missense mutation in combination with a null mutation [6]. Recently, also patients with a homozygous deletion, causing the loss of a single amino acid, were reported [14]. These mutations all lead to reduced protein stability and reduced enzyme capacity. None of the reported patients were totally devoid of enzyme activity. Altogether, this indicates that a total loss of PGM3 is also likely to be lethal in humans.

The *PGM3* mRNA exists in four major transcript isoforms, variants 1 and 4 with 14, and variants 2 and 3 with 13 exons, respectively. In this report, we describe a patient with a homozygous, hypomorphic splice-modifying mutation in an intron of the *PGM3* gene. This is the first patient described with altered splicing in *PGM3,* enabling estimation of the minimum levels of PGM3 necessary for survival.

## Case presentation

### Clinical data

The patient was a girl born as the third child of healthy parents that are first cousins of Pakistani origin. At 25 days of age she presented with multiple abscesses, predominantly located in the head, neck and axillae. Abscesses resolved after local and oral systemic antibiotics. At 1–2 months of age she had moderate mucocutatenous *Candida* infections with thrush that responded to local treatment. Chronic diarrhea started at 3 months of age and continued with varying intensity during her entire life. Multiple food allergies were identified and her gastrointestinal symptoms improved after exclusion of milk, cereals and eggs from her diet. From four months of age and onwards she suffered from recurrent bacterial infections of the skin, airways and lungs requiring repeated periods of hospitalization, and several septic episodes with *Staphylococcus aureus* identified in blood cultures occured. She also had disseminated eczema of varying severity and significant failure to thrive.

When arriving in Sweden at two years of age the girl had severe growth retardation with a height that was 5 standard deviations below the mean and weight was 6 standard deviations below the mean for her age. She had moderately enlarged lymph nodes in the neck and axillae, eczematous skin, but no obvious musculoskeletal abnormalities or delayed neurocognitive development were observed. Vascular abnormalities were noted in the form of hypoplasia and total occlusion of the superior vena cava and the right brachiocephalic vein. Multiple venous collateral vessels allowed venous return from the upper half of the body through the hemiazygos and azygos veins. The anatomy and function of the heart was normal.

Clinical findings are summarized in Table 1, the results of the immunological investigations are described in Table 2 and in more detail in Additional file 1: Table S1. All serum immunoglobulin classes were elevated with the most marked increase in IgE. In addition, the main abnormalities were a low total lymphocyte count and low CD3^+^ T-cell numbers together with reduced levels of T-cell receptor excision circles (TRECs), and low total number and ratio of naïve CD4^+^ and CD8^+^ T cells. A predominance of Th2 T cells (FITMAN panel) and impaired in vitro T cell proliferation in response to mitogens and antigens were also seen. Hyper-IgE syndrome was considered and whole genome sequencing was carried out, see below.

**Table 1:** Clinical data

Origin	Pakistan
Sex	Female
Age at last evaluation	3 years
Age at onset of symptoms	3 weeks
Anemia	+ (recurrent transfusions)
Abscesses/skin infections	+ (Multiple)
Bronchiectasis	–
Eczema/dermatitis	+ (Severe)
Otitis media	–
GI problems/food allergy	+ (Severe)
Pneumonia	+ (Multiple)
Encephalitis	–
Recurrent *Staph A* infections	+ (from 1 month of age)
Candida infections	+ (from 2 months of age)
Severe viral infections	+ (CMV)
Autoimmunity	+ (TSH receptor and TPO autoantibodies^a^)
Skeletal dysplasia	–
Scoliosis	–
Dysmorphic facial features	–
Developmental delay	–
Psychomotor retardation	–
Failure to thrive	+ (length -5SD below the mean, weight − 6 SD below the mean)
Hematopoietic stem cell transplantation	–
Splenomegaly	–
Vascular abnormalities	+ (hypoplasia/occlusion of superior vena cava)

^a^*TSH* Thyroid stimulating hormone, *TPO* Thyroperoxidase

**Table 2:** Selected laboratory data^a^

Analysis	Ref interval	Patient, at 24 months	Unit
Platelets	150–350	**456**	× 10^9^ /L
Eosinophils	0.04–0.4	**0.67**	× 10^9^ cells/L
IgG	3.5–10.5	**15**	g/L
IgA	0.07–0.55	**3.0**	g/L
IgM	0.27–1.2	**1.7**	g/L
IgE	< 13	**12,000**	kU/L
TREC (T-cell receptor excision circles)	> 1500	*1020*	*molecules/million cells*
In vitro T cell proliferation (PHA, ConA, PPD, candida)		*All low*	% of donors
FASCIA (T cell recall response, PHA, PWM, PPD, TT, candida, influenza, CMV, HSV, VZV)		*All low for both CD4* ^*+*^ *and CD8* ^*+*^	% of donors
Anti TSH receptor ab		*+*	
Anti TPO ab		*+*	
Absolute lymphocyte number	1.7–6.9	*0.94*	× 10^9^ cells/L
CD3^+^cells T cells	0.9–4.5	*0.60*	× 10^9^ cells/L
CD3^+^CD4^+^	0.62–0.86	*0.39*	× 10^9^ cells/L
CD3^+^CD4^+^CCR7^+^45RA^+^ Naïve CD4^+^T cells	0.26–0.38	*0.05*	× 10^9^ cells/L
CD3^+^CD4^+^CCR7^+^45RA^+^CD31^+^ CD4^+^ Recent thymic emigrants	0.12–0.24	*0.03*	× 10^9^ cells/L
CD3^+^CD4^+^CCR7^+^45RA^−^ Central memory CD4^+^ T cells	0.20–0.34	*0.13*	× 10^9^ cells/L
CD3^+^CD4^+^CCR7^+^45RA^−^CD38^+^HLA-DR^+^ Activated central memory CD4^+^ T cells	0–1	**4**	× 10^9^ cells/L
CD3^+^CD4^+^CCR7^−^45RA^−^CD38^+^HLA-DR^+^ Activated effector memory CD4^+^ T cells	0–0.01	**0.05**	× 10^9^ cells/L
CD3^+^CD4^+^CCR7^+^45RA^−^CXCR3^−^CCR6^+^ TH17 cells	0.06–0.10	*0.02*	× 10^9^ cells/L
CD3^+^CD4^+^CCR4^+^CD25hiCD127low45RO^−^ Naïve Treg	0.02–0.02	*< 0.01*	× 10^9^ cells/L
CD3^+^CD8^+^	0.25–0.49	*0.18*	× 10^9^ cells/L
CD3^+^CD8^+^CCR7^+^45RA^+^ Naïve CD8+ T cells	0.07–0.13	*< 0.01*	× 10^9^ cells/L
CD3^+^CD8^+^CCR7^+^45RA^−^ Central memory CD8+ T cells	0.03–0.05	*< 0.01*	× 10^9^ cells/L
CD3^+^CD8^+^CCR7^−^45RA^−^CD38^+^HLADR^+^ Activated effector memory CD8^+^ T cells	0–0.01	**0.05**	× 10^9^ cells/L

Abbreviations found in the table: *CMV* Cytomegalo virus, *ConA* Concanavalin A, *HSV* Herpes simplex virus, *PHA* Phytohaemagglutinin, *PPD* Tuberculin antigen, *PWM* Pokeweed mitogen, *TT* Tetanus toxoid, *VZV* Varicella zoster virus, *TSH* Thyroid stimulating hormone, *TPO* Thyroperoxidase

^a^Selected laboratory data with numbers outside of reference intervals, for a complete list of performed analyses see Additional file 1: Table S2. Elevated levels in bold, reduced levels in italic

Treatment with allogeneic hematopoietic stem cell therapy was planned, but unfortunately her clinical condition deteriorated with disseminated scalding skin infections, reactivation of cytomegalovirus and multi-organ failure, which led to her death at 3 years of age.

### Identification of the PGM3 mutation

**Fig. 1:**
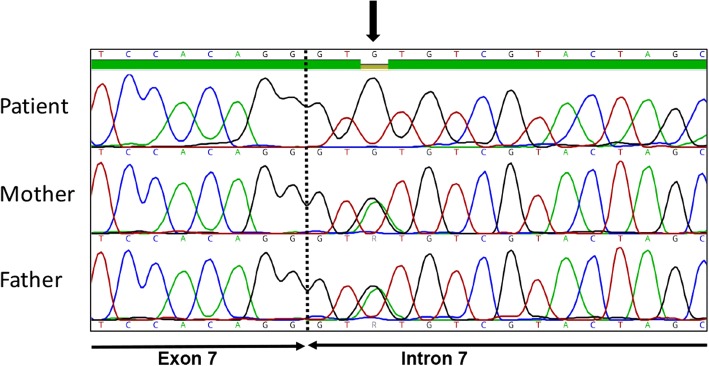
Sanger sequencing verifying the PGM3 variant. Chromatograms from the patient and her parents are shown. The arrow indicates the mutation (A > G)

Genomic DNA was prepared and used for sequencing as described in the Additional file 1: Supplementary methods. Bioinformatic analysis was carried out using v1.5.6 of Mutation Identification Pipeline, MIP [15]. Sequence reads were aligned to the whole human genome reference GRCh37, and visualized through the browser-based software Scout, as described in the Additional file 1: Supplementary material and methods. The clinical interpretation of sequence variants was restricted to a pre-defined list of 305 immunodeficiency genes (Additional file 1: Table S2). After filtering on variant functional annotation, allele frequency and phenotypic associations, a homozygous splice-region variant in the *PGM3* gene remained as a plausible candidate. The variant, an adenine to guanine substitution on chromosome 6; position 83,891,452 (c.871 + 3 A > G in transcript NM_001199917), affected the third base in intron 7 of the *PGM3* gene and was absent from public databases (1000Genomes and ExAC). No other missense or splice variants were detected in the *PGM3* gene, nor in the other genes associated with Hyper-IgE. The sole exception was a heterozygous missense variant in *DOCK8* (c.459C > A; p.Asp153Glu). This variant, however, received a low rank score by MIP since it had been classified as benign and tolerated using the SIFT [16] and Polyphen [17] data tools, respectively, and was reported to be of “uncertain significance” in ClinVar [18]. Furthermore, it had a population frequency of 2/1000 in ExAC [19] and lacked a putative compound variant. It was not investigated further. The *PGM3* variant was verified by Sanger sequencing of DNA from the patient, and her mother and father. Both parents were heterozygous for the mutation (Fig. 1).

### Identification of new *PGM3* splice isoforms

We then investigated whether the identified mutation in intron 7 would influence splicing of the pre-mRNA and thus PGM3 protein production. To minimize blood sampling, Epstein-Barr Virus (EBV) transformation of cells from the patient and healthy controls was carried out as described in Additional file 1: Supplementary methods. RNA was extracted from peripheral blood and EBV transformed cells and cDNA was subsequently prepared using random hexamer primers. Reactions were performed with primers as indicated in figures, for primer sequences and PCR protocols see Additional file 1: Supplementary methods.

**Fig. 2:**
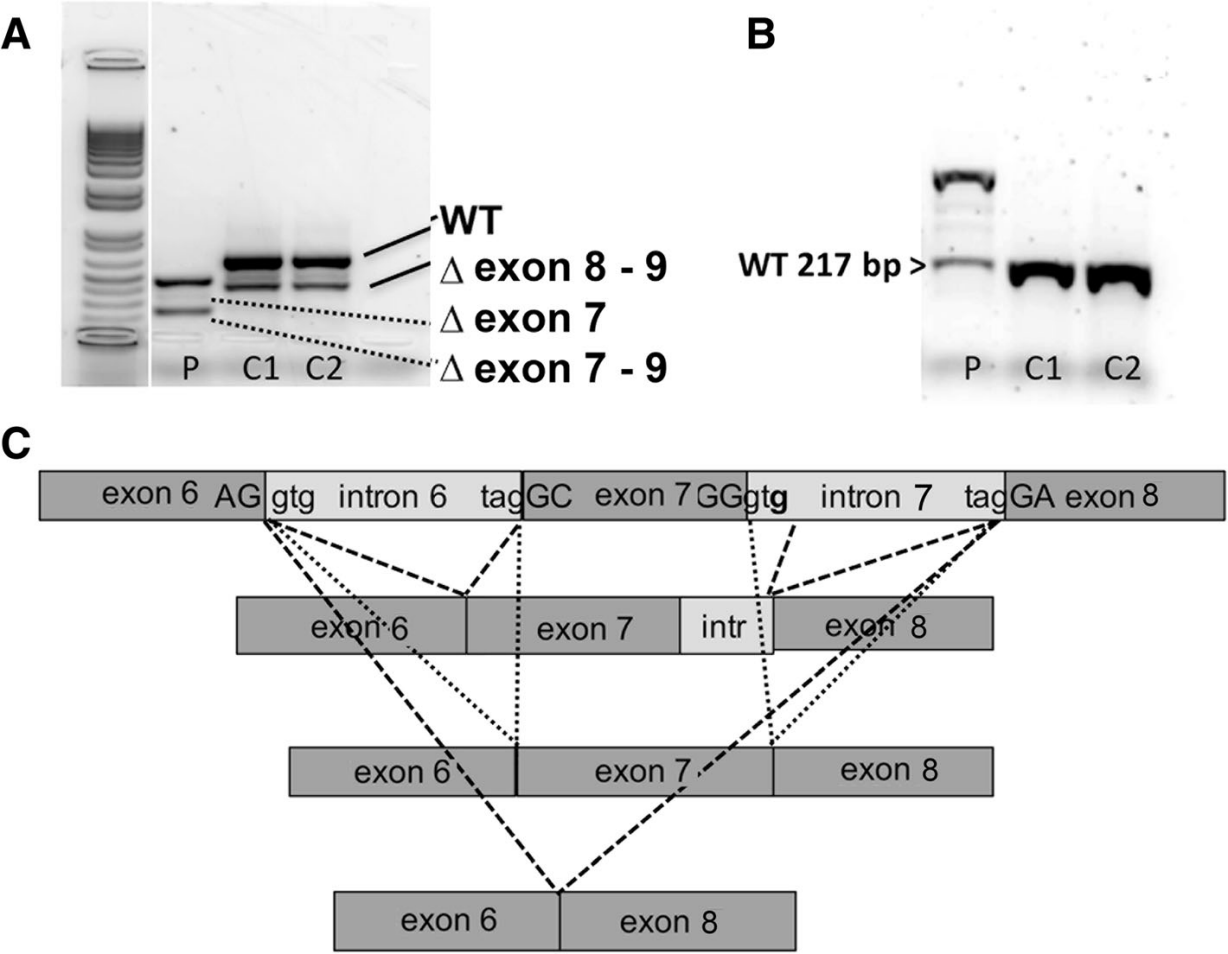
RT-PCR performed on mRNA from peripheral blood and EBV-transformed B-cells. Agarose gel analysis of RT-PCR performed on cDNA from patient cells (P) and from two healthy controls (C1 and C2). Fragments were purified from gel and analyzed by Sanger sequencing. The PCRs were performed using **a**) forward primer in exon 5 and reverse primer in exon 10, **b**) forward primer in exon 7 and reverse primer in exon 8, **c**) A schematic illustration of splice events found in the patient samples

Using forward primers binding in exon 4 or 5 and reverse primers in exon 10, two bands were seen in both controls and patient samples, (Fig. 2a). Sequencing revealed that the PCR-fragments from controls corresponded to full-length cDNA and a species missing exon 8 + 9 respectively (Additional file 1: Figure S1A), while no full-length mRNA was found in the patient samples. The deletion of exon 8 and 9 removes 242 bases. We have not found any registered PGM3 mRNAs missing this sequence. The deletion results in premature stops in all reported mRNAs and can thus not be considered to have any biological relevance. Patient mRNA species were missing exon 7 only, or exons 7–9 (Additional file 1: Figure S1B). The loss of the whole exon 7 removes 196 bases which also induces a frame shift. Because all four major *PGM3* isoforms contain exons 7, 8 and 9, the splice-mutation as well as the shorter species in the control samples could not be related to a specific isoform.

We then designed primers, binding within exons 7 and 8. This resulted in a single band for the control and, somewhat unexpectedly, two fragments for the patient cDNA (Fig. 2b). Sequencing showed that the smaller fragment corresponds to a correctly spliced mRNA, while the larger fragment contained additional sequences from intron 7 (+ 462 bases) (for sequences see Additional file 1: Figure S2 A and B). Even though the species with partial intron retention does not induce a frame shift, it contains stop codons and can thus not be translated to an intact protein. A schematic illustration of the splicing events for patient mRNA is shown in Fig. 2c. Using primers, which both bind further downstream of the mutation, resulted in fragments of the same size for both control and patient samples (Additional file 1: Figure S3).

**Fig. 3:**
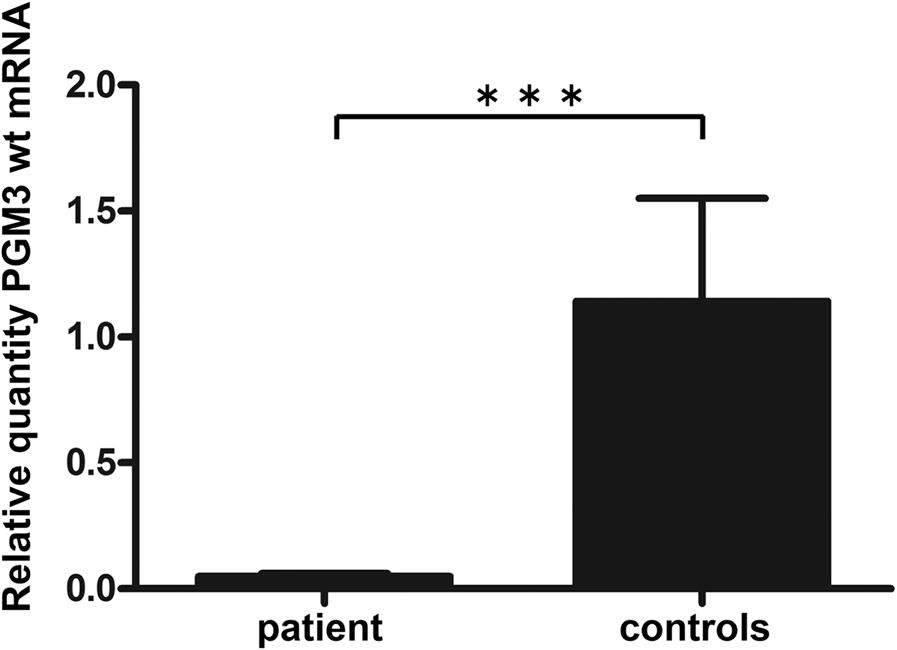
Relative *PGM3* mRNA levels in EBV-transformed B-cells from patient and healthy controls. The figure shows the mean for patient cells harvested at different time-points (*n* = 4) and for the mean of four healthy controls harvested at three time-points. *PGM3* mRNA in total RNA as quantified to *HPRT* mRNA using real time RT-PCR and the ΔΔCT method. Error-bars show standard deviations of the mean. *** *p* < 0.001 calculated by two-tailed Student’s t-test

Since all *PGM3* isoforms contain exon 7 and 8 and the RNA species lacking exon 8 and 9 is not resulting in a known protein isoform, we placed the primers for the q-PCR in such a way that the quantification only reflects correctly spliced transcripts containing exons 7 and 8. The forward primer was designed to bind exactly over the exon 7/8 junction while the reverse primer was binding in exon 8. Quantitative real time RT-PCR, showed that the amount of correctly spliced mRNA was only around 5% of the mean level found in four healthy controls (Fig. 3).

### Quantification of PGM3 protein levels

**Fig. 4:**
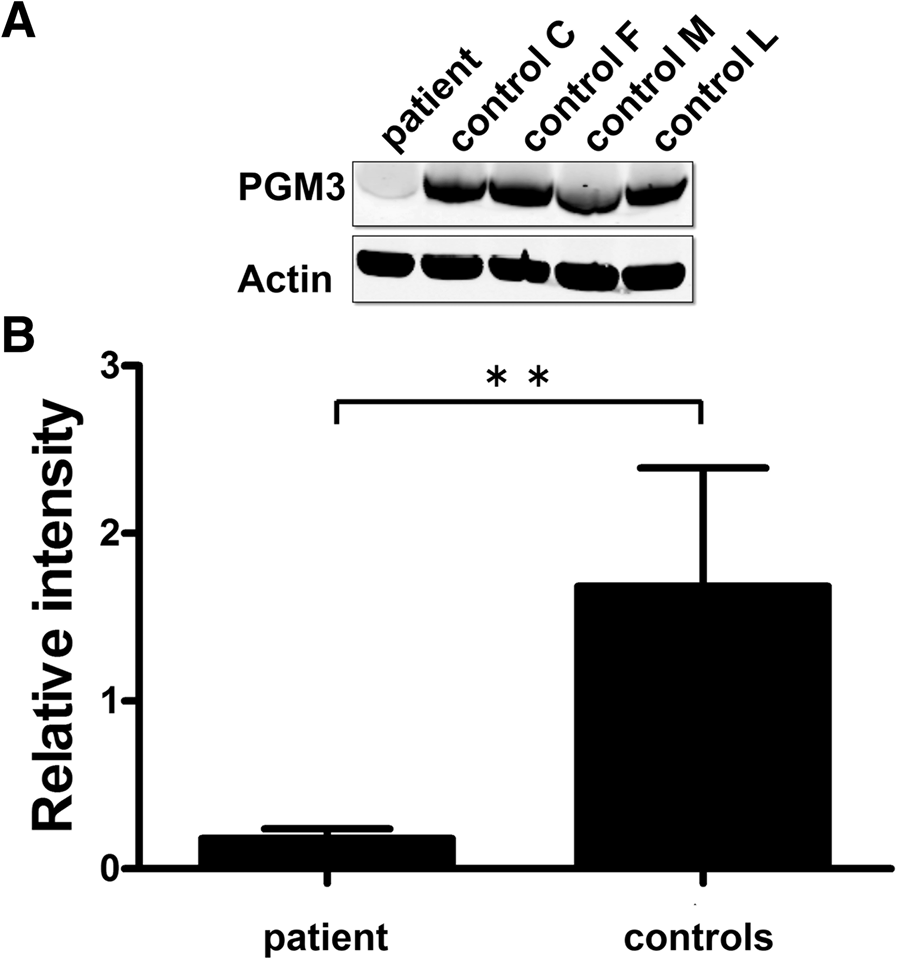
Relative PGM3 protein levels in EBV-transformed B-cells from patient and healthy controls. Western blot performed on whole cell lysate from EBV-transformed B-cells. A blot from a representative experiment is shown at the top. Quantification of PGM3 levels relative to actin levels as mean from three different experiments is shown in the lower panel. Error bars represents standard deviation of the mean. The control is shown as the mean of values from four healthy controls. *p* ≤ 0.0038 calculated by two-tailed Student’s t-test

To analyze the intracellular PGM3 protein levels, whole cell lysates prepared from EBV cells established from the patient and healthy controls were analyzed by Western blot (Fig. 4). Staining for specific proteins was carried out using rabbit anti-PGM3 and mouse anti-actin antibodies, quantified and PGM3 relative to actin levels were calculated (see Additional file 1: Supplementary methods).

We found that the level of PGM3 protein present in the patient cells did not exceed 11% of the mean level in four healthy controls.

## Discussion and conclusions

There are still no established phenotypic markers for PGM3 deficiency, and the number of reported patients is low. In most of the described cases the patients present a phenotype found in glycosylation disorders, including neurologic defects, dysmorphic features, and malformations. In the majority of PGM3 deficient patients very high serum IgE is also found [4, 5, 11]. However, while many patients develop severe immunodeficiency in combination with high serum IgE levels there are cases where patients lack this serological marker [7]. Most PGM3 deficient patients also display severe eczema, but recently two patients with severe immunodeficiency without eczema were described [9]. The patient described in this report, to our knowledge the first case with a hypomorphic splice-altering mutation in the *PGM3* gene, also displayed highly elevated serum IgE in combination with immune abnormalities. Still, unlike many of the other described patients this girl did not show any skeletal dysplasia or delayed neurocognitive development. In a few of the recently described cases, cardiovascular changes were reported [8, 9], and cardiovascular abnormalities in combination with hyper-IgE have been described earlier in STAT3 deficient patients [20]. In our patient, vascular abnormalities were found but no heart malformations. In addition, she suffered from a severe, mainly T-cell related, immunodeficiency. While earlier described cases suffered from severe combined B and T cell deficiency [6, 8, 9], the number and distribution of B cells in this patient were within the normal range, although total CD3^+^ T cells were reduced in number. T cell proliferation and responses to recall antigens were also diminished despite increased levels of activated memory T cells. Notably, both the total level and the ratio of recent thymic emigrants, naïve Treg, naïve CD4^+^ and CD8^+^ T cells and Th17 cells were reduced.

The mutation identified here was not found in any of the public databases 1000G and ExAC, and was not present either in a local database of 638 sequenced patients. The region seems well covered both in ExAc and gnomAD. Thus, poor coverage in this region cannot explain that this mutation was not identified earlier. While unlikely, since we do not have access to a collection of DNA samples from a Pakistani control population, we cannot on statistical grounds rule out the possibility that the identified splice site mutation exists as a normal variant in this population. Analyzing the mRNA by RT-PCR confirmed miss-splicing associated with the presence of a single base substitution (A > G) in position + 3 of intron 7. Finding a G in + 3 position of an intron 5′ splice site, as in the patient sequence is not unusual. However, the wild type sequence of the 5′ splice site (ACAGG/GUAUGUCG) is still relatively uncommon due to the U in intron position + 4 [21]. Beside the actual splice-site motif, splicing is influenced by additional sequences within exons as well as in introns, so called splice enhancer (SSE) or splice silencing sequences. As can be seen in Additional file 1: Figure S4, the mutation increases the number of exonic splice silencing sites in this exon/intron border. The mutated splice-site is now so weak that an alternative cryptic splice site further down in intron 7 is also used. Similar erroneous splicing events have been reported in X-linked agammaglobulinemia for instance, where an intact splice-site has been skipped and replaced by an intronic cryptic, suboptimal splice-site due to a change in a SSE sequence [22].

It is always hard to predict the in vivo capacity of mutated enzymes due to a combination of reduced activity and stability. Recombinant PGM3 enzymes with identified mutations have been tested in in vitro enzyme assays [6, 7]. For one of these mutations the enzyme was shown to have around 50% specific enzyme activity while still causing immunodeficiency in the homozygous patient, possibly due to protein instability. Earlier reports of patients with 50% residual enzyme activity might also be overestimated due to in vitro analysis methods, since heterozygous carriers with a null mutation in the PGM3 gene are healthy [6]. It should also be kept in mind that while the existing in vitro assays provide information, it is not known whether they reflect the natural activity of PGM3 in humans. In the patient reported here, the only existing PGM3 protein is derived from the fraction of correctly spliced mRNA, yielding a stable and fully functional wild type protein. Thus, it was for the first time possible to calculate the residual enzyme activity in vivo. By quantitative RT-PCR the wild type mRNA level was found to be 5% of the level in controls. However, according to the Western blot protein quantification the total amount of protein was around 11% of the level found in healthy controls (*n* = 4). Although mRNA quantification is more precise, there is not always a conserved ratio between mRNA and protein levels. It is known that protein levels can be “buffered” to maintain a stable intracellular level [23], compatible with the higher ratio found in the patient cells.

There is still no specific phenotype identified that distinguishes immunodeficiency caused by *PGM3* mutations from other forms of immunodeficiency. Moreover, the phenotype differs considerably among affected patients. Thus, apart from providing insight into the enzyme levels needed for survival, the patient described here yields new information on the phenotypic variability among patients.

## Additional file

Additional file 1: **Table S1.** Lab data (Nov 2015, age 24 months). **Table S2.** Current Gene list for Congenital immune defects (*n*=305). **Figures S1A and B.** Alignment of sequences for PCR products from patient and control samples. **Figures S2 A and B.** Alignment of the sequence of PCR fragments from patient samples using a forward primer binding in exon 7 and the reverse primer binding in exon 8. **Figures S3 A and B.** RT-PCR performed on mRNA from peripheral blood and EBV-transformed B-cells. **Figure S4.** Identified exonic splicing silencer(ESS) and intronic splicing enhanser (ISE) sequences around the mutation site. Material and methods. (PDF 3420 kb)

## Abbreviations

*DOCK8*: Dedicator of cytokinesis 8
EBV: Epstein-Barr Virus
GlcNAc: N-acetylglucosamine
MIP: Mutation Identification Pipeline
PGM3: Phosphoglucomutase 3
STAT3: Signal transducer and activator of transcription 3
TYK2: Tyrosine kinase 2
UDP-GlcNac: Uridine diphosphate N-acetylglucosamine
