# Supplementary material for: Eleven percent intact PGM3 in a severely immunodeficient patient with a novel splice-site mutation, a case report
Source: BMC Pediatr. 2018 Aug 29;18:285. doi: 10.1186/s12887-018-1258-9 (PMC6114780; doi:10.1186/s12887-018-1258-9)
Supplement: Supplementary file 1 — Table S1. Lab data (Nov 2015, age 24 months). Table S2. Current Gene list for Congenital immune defects (n=305). Figures S1A and B. Alignment of sequences for PCR products from patient and control samples. Figures S2 A and B. Alignment of the sequence of PCR fragments from patient samples using a forward primer binding in exon 7 and the reverse primer binding in exon 8. Figures S3 A and B. RT-PCR performed on mRNA from peripheral blood and EBV-transformed B-cells. Figure S4. Identified exonic splicing silencer(ESS) and intronic splicing enhanser (ISE) sequences around the mutation site. Material and methods. (PDF 3420 kb) [file 12887_2018_1258_MOESM1_ESM.pdf]

Additional information

Table S1

Lab data (age 24 months):

	Unit	24 mo, Nov 2015	Ref interval
Hemoglobin	g/L	81	105 - 135
Platelets	$x 10^9$ /L	456	150 - 350
Leucocytes	$x 10^9$ cells/L	5.9	6.0 - 17
Neutrophils	$x 10^9$ cells/L	3.5	1.5 – 8.5
Eosinophils	$x 10^9$ cells/L	0.67	0.04 – 0.4
Basophils	$x 10^9$ cells/L	0.15	0 - 0.1
Monocytes	$x 10^9$ cells/L	0.50	0.1 – 1.0
IgG	g/L	15	3.5 – 10.5
IgA	g/L	3.0	0.07 – 0.55
IgM	g/L	1.7	0.27 – 1.2
IgE	kU/L	12,000	<13
TREC	molecules/miljon cells	1,020	>1,500
CMV IgG		+	
CMV IgM		+	
CMV PCR		+	
EBV IgG		+	
EBV IgM		-	
EBV PCR		-	
In vitro T cell proliferation (PHA, ConA, PPD, candida)	% of donors	All low	
FASCIA (T cell recall response, PHA, PWM, PPD, TT, candida, influenza, CMV, HSV, VZV)	% of donors	All low CD4+ and CD8+	
Complement screening (classical/alternative)		Normal x 2	
Phagocyte function (DHR assay)		Normal	
Anti-TSH receptor ab		+	
Anti-TPO ab		+	
TSH and thyroid hormones		normal	
**Immunophenotype (modified FITMAN-panel)**			
Absolute lymphocyte number	$x 10^9$ cells/L	0.94	1.7 – 6.9
CD3+ cells<br>T cells	$x 10^9$ cells/L	0.60	0.9 – 4.5
CD3+ cells<br>T cells	% of lymphocytes	64	43 - 76
CD3-CD19+CD20+<br>B cells	$x 10^9$ cells/L	0.18	0.16 – 2.0
CD3-CD19+CD20+<br>B cells	% of lymphocytes	20	9 - 24
CD3-CD56+<br>NK cells	$x 10^9$ cells/L	0.13	0.1 – 1.0
CD3-CD56+<br>NK cells	% of lymphocytes	14	4 - 23
**CD4+ cells**			
CD3+CD4+	$x 10^9$ cells/L	0.39	0.62 – 0.86
CD3+CD4+	% of CD3+	65	58 - 70
CD3+CD4+CCR7+45RA+	$x 10^9$ cells/L	0.05	0.26 – 0.38

Naïve CD4+ T cells			
CD3+CD4+CCR7+45RA+<br>Naïve CD4+ T cells	% of CD3+	9	22 - 34
CD3+CD4+CCR7+45RA+CD31+<br>CD4+ Recent thymic emigrants	x 10^9 cells/L	0.03	0.12 – 0.24
CD3+CD4+CCR7+45RA+CD31+<br>CD4+ Recent thymic emigrants	% of CD3+	5	11 - 19
CD3+CD4+CCR7+45RA-<br>Central memory CD4+ T cells	x 10^9 cells/L	0.13	0.20 – 0.34
CD3+CD4+CCR7+45RA-<br>Central memory CD4+ T cells	% of CD3+	21	18 - 27
CD3+CD4+CCR7-45RA+<br>Effector CD4+ T cells	x 10^9 cells/L	<0.01	0 – 0.02
CD3+CD4+CCR7-45RA+<br>Effector CD4+ T cells	% of CD3+	1	0 - 1
CD3+CD4+CCR7-45RA-<br>Effector memory CD4+ T cells	x 10^9 cells/L	0.19	0.11 – 0.22
CD3+CD4+CCR7-45RA-<br>Effector memory CD4+ T cells	% of CD3+	21	11 - 15
CD3+CD4+CCR7+45RA+CD38+HLADR+<br>Activated naïve CD4+ T cells	x 10^9 cells/L	<0.01	0 – 0.01
CD3+CD4+CCR7+45RA+CD38+HLADR+<br>Activated naïve CD4+ T cells	% of CD3+	<1	0 - 1
CD3+CD4+CCR7+45RA-CD38+HLADR+<br>Activated central memory CD4+ T cells	x 10^9 cells/L	4	0 - 1
CD3+CD4+CCR7+45RA-CD38+HLADR+<br>Activated central memory CD4+ T cells	% of CD3+	0.02	0 - 1
CD3+CD4+CCR7-45RA+CD38+HLADR+<br>Activated effector CD4+ T cells	x 10^9 cells/L	<0.01	0 - 1
CD3+CD4+CCR7-45RA+ CD38+HLADR+<br>Activated effector CD4+ T cells	% of CD3+	<1	0 - 1
CD3+CD4+CCR7-45RA-CD38+HLADR+<br>Activated effector memory CD4+ T cells	x 10^9 cells/L	0.05	0 – 0.01
CD3+CD4+CCR7-45RA-CD38+HLADR+<br>Activated effector memory CD4+ T cells	% of CD3+	8	0.4 – 0.7
CD3+CD4+CCR7-45RAlowCD38+HLADR+<br>CD4+ EMRA	x 10^9 cells/L	<0.01	0 - 1
CD3+CD4+CCR7-45RAlowCD38+HLADR+<br>CD4+ EMRA	% of CD3+	0.26	0 – 0.02
CD3+CD4+CCR7+45RA-CXCR3+CCR6-<br>TH1 cells	x 10^9 cells/L	0.14	0.13 - 0.21
CD3+CD4+CCR7+45RA-CXCR3+CCR6-<br>TH1 cells	% of CD3+	24	12 - 16
CD3+CD4+CCR7+45RA-CXCR3-CCR6-<br>TH2 cells	x 10^9 cells/L	0.20	0.30 – 0.42
CD3+CD4+CCR7+45RA-CXCR3-CCR6-<br>TH2 cells	% of CD3+	34	27 - 37
CD3+CD4+CCR7+45RA-CXCR3-CCR6+<br>TH17 cells	x 10^9 cells/L	0.02	0.06 – 0.10
CD3+CD4+CCR7+45RA-CXCR3-CCR6+<br>TH17 cells	% of CD3+	4	5.0 – 7.6
CD3+CD4+CCR4+CD25hiCD127low45RO-<br>Naïve Treg	x 10^9 cells/L	<0.01	0.02 – 0.02
CD3+CD4+CCR4+CD25hiCD127low45RO-<br>Naïve Treg	% of CD3+CD4+	<1	2.4 – 3.8

CD3+CD4+CCR4+CD25hiCD127low45RO+<br>Memory Treg	x 10^9 cells/L	0.03	0.02 – 0.04
CD3+CD4+CCR4+CD25hiCD127low45RO+<br>Memory Treg	% of CD3+CD4+	8	3.4 – 5.4
CD3+CD4+CCR4+CD25hiCD127low45RO-HLADR+<br>Activated naïve Treg	x 10^9 cells/L	<0.01	0 - 1
CD3+CD4+CCR4+CD25hiCD127low45RO-HLADR+<br>Activated naïve Treg	% of CD3+CD4+	<1	0 – 0.6
CD3+CD4+CCR4+CD25hiCD127low45RO+HLADR+<br>Activated memory Treg	x 10^9 cells/L	0.02	0.01 – 0.02
CD3+CD4+CCR4+CD25hiCD127low45RO+HLADR+<br>Activated memory Treg	% of CD3+CD4+	6	1.3 – 2.1
**CD8+ cells**			
CD3+CD8+	x 10^9 cells/L	0.18	0.25 – 0.49
CD3+CD8+	% of CD3+	30	24 - 34
CD3+CD8+CCR7+45RA+<br>Naïve CD8+ T cells	x 10^9 cells/L	<0.01	0.07 – 0.13
CD3+CD8+CCR7+45RA+<br>Naïve CD8+ T cells	% of CD3+	1	6 – 12
CD3+CD8+CCR7+45RA-<br>Central memory CD8+ T cells	x 10^9 cells/L	<0.01	0.03 – 0.05
CD3+CD8+CCR7+45RA-<br>Central memory CD8+ T cells	% of CD3+	<1	2.6 – 5.0
CD3+CD8+CCR7-45RA+<br>Effector CD8+ T cells	x 10^9 cells/L	0.05	0.01 – 0.13
CD3+CD8+CCR7-45RA+<br>Effector CD8+ T cells	% of CD3+	8	2 – 8
CD3+CD8+CCR7-45RA-<br>Effector memory CD8+ T cells	x 10^9 cells/L	0.09	0.06 – 0.10
CD3+CD8+CCR7-45RA-<br>Effector memory CD8+ T cells	% of CD3+	15	5 - 8
CD3+CD8+CCR7+45RA+CD38+HLADR+<br>Activated naïve CD8+ T cells	x 10^9 cells/L	<0.01	0 – 1
CD3+CD8+CCR7+45RA+CD38+HLADR+<br>Activated naïve CD8+ T cells	% of CD3+	<1	0 – 0.03
CD3+CD8+CCR7+45RA-CD38+HLADR+<br>Activated central memory CD8+ T cells	x 10^9 cells/L	<0.01	0 – 1
CD3+CD8+CCR7+45RA-CD38+HLADR+<br>Activated central memory CD8+ T cells	% of CD3+	<1	0 – 1
CD3+CD8+CCR7-45RA+CD38+HLADR+<br>Activated effector CD8+ T cells	x 10^9 cells/L	0.02	0 – 0.01
CD3+CD8+CCR7-45RA+CD38+HLADR+<br>Activated effector CD8+ T cells	% of CD3+	4	0 – 1
CD3+CD8+CCR7-45RA-CD38+HLADR+<br>Activated effector memory CD8+ T cells	x 10^9 cells/L	0.05	0 – 0.01
CD3+CD8+CCR7-45RA-CD38+HLADR+<br>Activated effector memory CD8+ T cells	% of CD3+	8	0 - 1
CD3+CD8+CCR7-45RAlowCD38+HLADR+<br>CD8+ EMRA	x 10^9 cells/L	0.01	0 – 0.01
CD3+CD8+CCR7-45RAlowCD38+HLADR+<br>CD8+ EMRA	% of CD3+	2	0 – 1
**B cells**			
CD3-CD19+CD20+CD27-<br>CD24lowCD38lowIgD+IgM+<br>Naïve B cells	x 10^9 cells/L	0.10	0.05 – 1.0

CD3-CD19+CD20+CD27-<br>CD24lowCD38lowIgD+IgM+<br>Naïve B cells	% of CD19+	56	32 – 90
CD3-CD19+CD20+CD27-CD24hiCD38hiIgD+IgM+<br>Transitional B cells	x 10^9 cells/L	0.03	0.01 – 0.05
CD3-CD19+CD20+CD27-CD24hiCD38hiIgD+IgM+<br>Transitional B cells	% of CD19+	16	4 - 30
CD3-CD19+CD20+CD27+IgD+IgM+<br>Marginal zone-like B cells	x 10^9 cells/L	<0.01	0.01 – 0.25
CD3-CD19+CD20+CD27+IgD+IgM+<br>Marginal zone-like B cells	% of CD19+	2	2 -20
CD3-CD19+CD20+CD27+IgD-IgM+<br>IgM+ memory B cells	x 10^9 cells/L	<0.01	0 – 0.02
CD3-CD19+CD20+CD27+IgD-IgM+<br>IgM+ memory B cells	% of CD19+	2	1 - 5
CD3-CD19+CD20+CD27+IgD-IgM-<br>Class switched memory B cells	x 10^9 cells/L	<0.01	0.01 – 0.05
CD3-CD19+CD20+CD27+IgD-IgM-<br>Class switched memory B cells	% of CD19+	4	1 – 20
CD3-CD19+CD20-CD38+CD27hi<br>Plasmablasts	x 10^9 cells/L	0.01	0 – 0.04
CD3-CD19+CD20-CD38+CD27hi<br>Plasmablasts	% of CD19+	6	0 - 5

Table S2. **Current Gene List for Congenital Immune Defects (N=305)**

IBTK
BTK
IGHM
IGLL1 (λ5)
CD79A
CD79B
BLNK
LRRC8A
ICOS
CD19
CD81
MS4A1 (CD20)
TNFRSF13B (TACI)
TNFRSF13C (BAFF-R)
CD21 / CR2
CD40LG
CD40
AICDA
UNG
CARD11/CARMA11/BIMP3
IL21R
LRBA
MOGS
SPPL2A
TCF3 / E2A
PIK3R1
TWEAK / TNFSF12
NFKB2
PRKCD
TRNT1
CLEC16A
IL21
NOTCH1
RTP4
MAP3K14
MEFV
MVK
CIAS1 / PYPAF1 /NLRP3
NALP12 /NLRP12
TNFRSF1A
IL10RA
IL10RB
IL10
PSTPIP1
NOD2
LPIN2
IL1RN
RBCK1 / HOIL1 / XAP3
IL36RN
SLC29A3
CARD14 (CARMA2)
SH3BP2
PSMB8
PLCG2
CECR1 / ADA2
TAP2
TAP1
TAPBP (Tapasin)
RFX5
RFXAP
MHC2TA / CIITA
RFXANK
ORAI1
STIM1
CD8A
ZAP70
RHOH
MSN
PIK3CD
CTPS1
IKBKB
TRAC
MALT1
OX40 / TNFRSF4
ZBTB24 (ICF2)
DOCK2
CTLA4
CD28
NEIL3
RIPK1
SEC16A
TFRC
MIR181A1
C1QA
C1QB
C1QC
C1S
C1R
C4A
C4B
C2
C3
C5
C6
C7
C8A
C8B
C8G
C9
SERPING1 / C1INH
CFB
CFD
PFC / CFP
CFI
HF1 / CFH
MBL2
MASP1
MASP2
COLEC11/CLK1
CD46
PIGA
CD59
FCN3
C1QBP
CFHR1
CFHR3
THBD
TPP2
IKBKG
NFKBIA
IRAK4
MYD88
CXCR4
TMC6 / EVER1
TMC8 / EVER2
STAT2
RPSA
PBX1
NLK
IRF7
MST1
DKC1
NOLA3 / NOP10
TERC

TINF2
NHP2
RTEL1
PRF1 (Perforin)
UNC13D
STX11
STXBP2
SH2D1A
XIAP
ITK
CD27 / TNFRSF7
ELANE
GFI1 / ZNF163
HAX1
G6PC3
SLC37A4
P14 / MAPBPIP / LAMTOR2
TAZ
COH1 / VPS13B
C16orf57 (USB1)
ITGB2 (CD18)
SLC35C1 / FUCT1 (Fucose transp.)
KIND3 / KINDLIN3 / FERMT3
RAC2
ACTB
FPR1
CTSC (Cathepsin C)
CEBPE
SBDS
CYBB / p91-PHOX
CYBA / p22-PHOX
NCF1 / p47-PHOX
NCF2 /p67-PHOX
NCF4 / p40-PHOX
MPO
GATA1
CSF3R
VPS45
JAGN1
CD4
TLR3
UNC93B1
TRAF3
TICAM1 / TRIF
TBK1
STAT3
DOCK8
TYK2
PGM3
UNC119
CHS1 / LYST
MYO5A
RAB27A
AP3B1
NKX2-5
MKL1
PTPN14
GJC2
PROX1
PIK3CA
VEGFC
RASA1
FLT4 / VEGFR3
GJA1
ITGA9
TSC1
PTEN
KIF11
FOXC2
TSC2
CCBE1
SOX18
G6PC (formerly G6PT)
ALG1
TCN2
SLC46A1
MTHFD1
IL12B/23 (p40)
IFNGR1
IFNGR2
IRF8
ISG15
IL12RB2
SLC11A1 / NRAMP1
SPPL2A
IL12RB1
STAT1
FCGR3A/CD16B
MCM4
INO80
GCCR / NR3C1
TNFSF11
TNFRSF11A
TCIRG1
OSTM1
CLCN7
SNX10
PLEKHM1
CA2
GATA2
GMCSFR / CSF2RA
CARD9
IL17RA
IL17F
TRAF3IP2 / ACT1
CLEC7A
APOL1
RMRP
IKZF1 (Ikaros)
STAT5B
MAGT1
WAS
WIPF1
VAV1
ATM
MRE11A
NBS1 / NBN
RECQL3 / BLM
DNMT3B
RNF168
SMARCAL1
SPINK5
SP110
CHD7
POLE
TTC7A
TPP1
TERT
LCK
PNP
STK4
TNFRSF6 (CD95/Fas)
TNFSF6 (CD178/FASLG)
CASP10
CASP8
NRAS
FADD
AIRE

FOXP3
IL2RA
ITCH
KRAS
MUC2
TMEM173
FOXN1
TBX1
IL2RG
JAK3
IL7R
PTPRC
CD3D
CD3E
CD3G
CD247
CORO1A
RAG1
RAG2
DCLRE1C
LIG4
LIG1
PRKDC
AK2
ADA
NHEJ1
TREX1
RNASEH2A
RNASEH2B
RNASEH2C
SAMHD1
ADAR
ACP

Figure S1

A

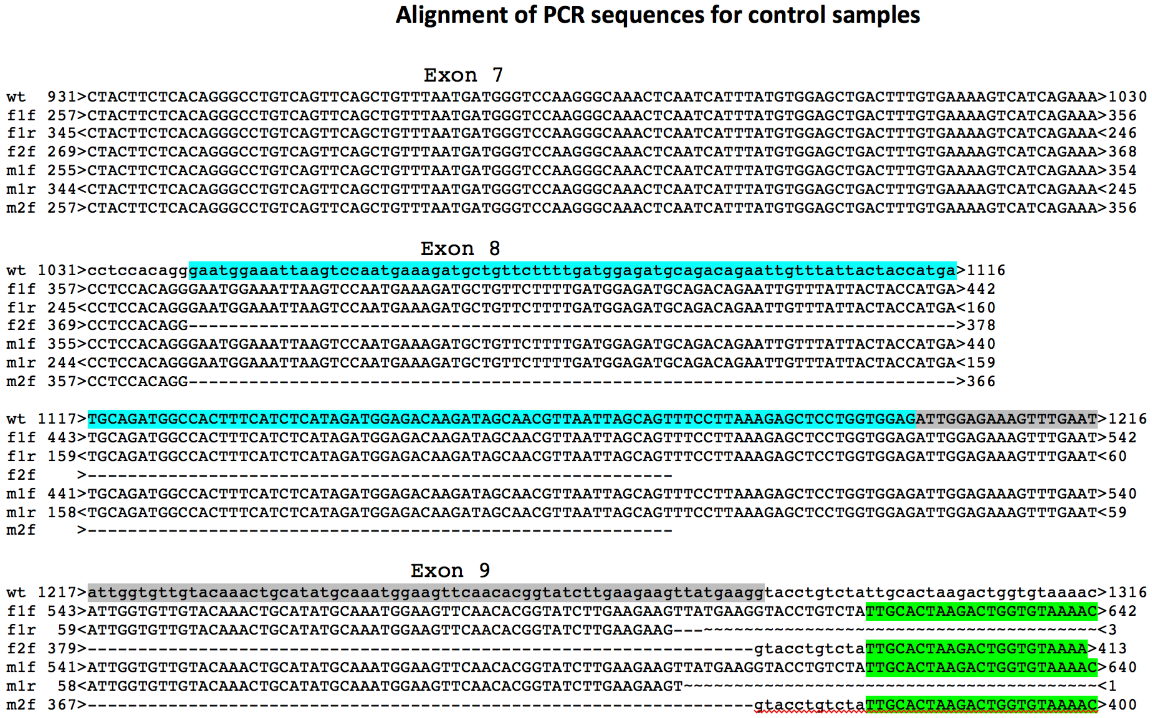

B

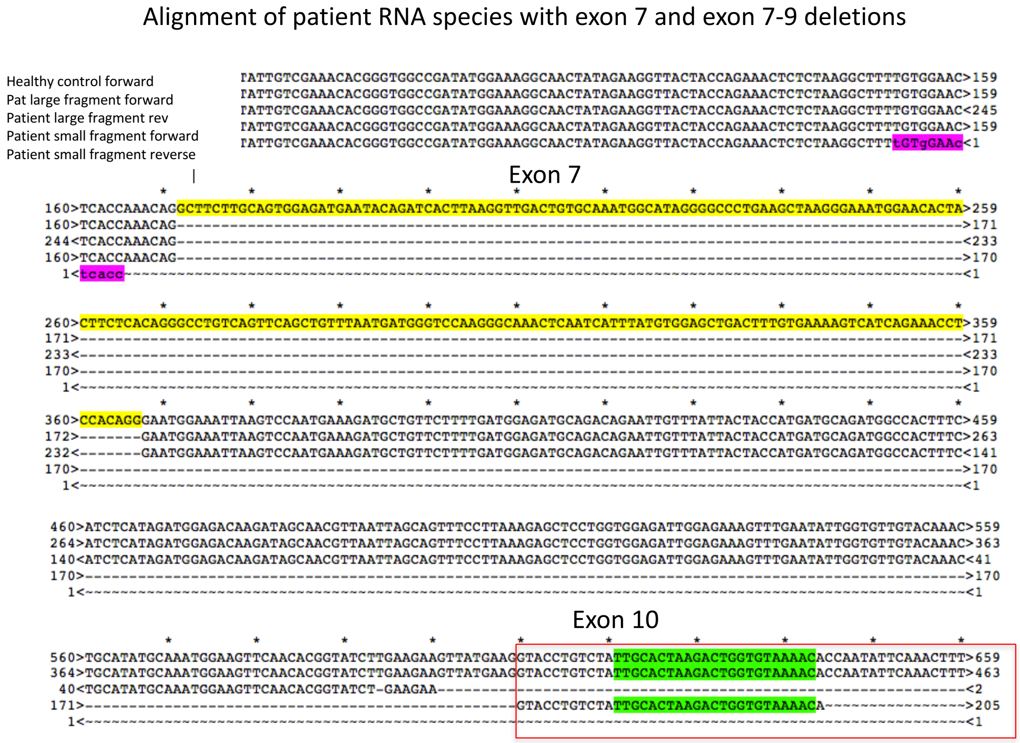

Figure S1. Alignment of sequences for PCR products from patient and control samples. A) PCR fragments from control samples aligned to the wt sequence, f1 and m1 are the larger and f2 andm2 the shorter fragments from the PCR for two healthy controls. B) PCR fragments from patient samples. Top row shows the wt mRNA sequence, rows 2 and 3 the large PCR fragment, rows 4 and 5 the sequences for the small fragment.

PCR was performed using a forward primer binding in exon 5 and the reverse primer binding in exon 10. Exon 7 in yellow, exon 8 in blue and exon 9 in gray. The position for the reverse primer is indicated in green.

Figure S2

A

Alignment of patient RNA species with partial intron 7 retention

Exon 7

```
WT       12101>ACAGGGCCTGTCAGTTCAGCTGTTTAATGATGGGTCCAAGGGCAAACTCAATCATTTATGTGGAGCTGACTTTGTGAAAAGTCATCAGAAACCTCCACAG>12200
Pat fw       1>~~~~~~~~~~~~~~~~~~~~~~~~~~~~~~~~~~~~~~~~~~~~~~~~~~~~~~~~~~~~~~~~~~~~~~~~~~~~~~~~~~~~ATCAGAAACCTCCACAG>16
Pat rev    632<-----------------------------------------------------CATTTATGTGGAGCTGACTTTGTGAAAAGTCATCAGAAACCTCCACAG<585

12201>GGTaTGTCGTACTAGCACTGCAAAAATTTGTTTTTAATGCTCTTTGAAGCCAATCTCACTTATAAACTAAGGTGGTAAAGTGAGTTAAGAATCTTGCAGC>12300
   17>GGTGTGTCGTACTAGCACTGCAAAAATTTGTTTTTAATGCTCTTTGAAGCCAATCTCACTTATAAACTAAGGTGGTAAAGTGAGTTAAGAATCTTGCAGC>116
  584<GGTGTGTCGTACTAGCACTGCAAAAATTTGTTTTTAATGCTCTTTGAAGCCAATCTCACTTATAAACTAAGGTGGTAAAGTGAGTTAAGAATCTTGCAGC<485

12301>TATTCCTGGCTCTGCCACATTTAGACATTGAGAAGTCTTCCCACTCTCAGAGCCATTGAAACATGGTCTGCTCTCTCTGCCTGTAAATCACACCATTCCT>12400
  117>TATTCCTGGCTCTGCCACATTTAGACATTGAGAAGTCTTCCCACTCTCAGAGCCATTGAAACATGGTCTGCTCTCTCTGCCTGTAAATCACACCATTCCT>216
  484<TATTCCTGGCTCTGCCACATTTAGACATTGAGAAGTCTTCCCACTCTCAGAGCCATTGAAACATGGTCTGCTCTCTCTGCCTGTAAATCACACCATTCCT<385

12401>CATAGTCTACAGGAAATCAGGTGGGGAAAGCAAAGTCAGGAAATGGCATTCTAGAGCAGTGGTTCTCAAACTGGCATGTGTCAGAATCACTTTGAGGGCT>12500
  217>CATAGTCTACAGGAAATCAGGTGGGGAAAGCAAAGTCAGGAAATGGCATTCTAGAGCAGTGGTTCTCAAACTGGCATGTGTCAGAATCACTTTGAGGGCT>316
  384<CATAGTCTACAGGAAATCAGGTGGGGAAAGCAAAGTCAGGAAATGGCATTCTAGAGCAGTGGTTCTCAAACTGGCATGTGTCAGAATCACTTTGAGGGCT<285

12501>TGTTAGCACACAGATTCCTGGGCTCCACTTCTGAGTTTCTGGAGGTCTGGCATAGGATTTGCATATGATAGAAATTTGCATTTCTATCAAGTTTGCAGGC>12600
  317>TGTTAGCACACAGATTCCTGGGCTCCACTTCTGAGTTTCTGGAGGTCTGGCATAGGATTTGCATATGATAGAAATTTGCATTTCTATCAAGTTTGCAGGC>416
  284<TGTTAGCACACAGATTCCTGGGCTCCACTTCTGAGTTTCTGGAGGTCTGGCATAGGATTTGCATATGATAGAAATTTGCATTTCTATCAAGTTTGCAGGC<185

12601>TTTGTTCCCAGTGCTTGTCTGGGGATCCCTTTTTGAGAATCACAGATCTACAGGGGAGCATAGTAAGTATTATTAGATACCTTATCTGAATTAAATACAC>12700
  417>TTTGTTCCCAGTGCTTGTCTGGGGATCCCTTTTTGAGAATCACAGATCTACAGGGGAGCATA--------------------------------------->479
  184<TTTGTTCCCAGTGCTTGTCTGGGGATCCCTTTTTGAGAATCACAGATCTACAGGGGAGCATA---------------------------------------<122
```

Exon 8

```
13901>AGAAAATTCTAAAACTCTTTATAATAAATTCTTAGACTGAAATGCATGTTAAATTATCCTGTGTGCTAGGAATGGAAATTAAGTCCAATGAAAGATGCTG>14000
  480>----------------------------------------------------------------------GAATGGAAATTAAGTCCAATGAAAGATGCTG>509
  121<----------------------------------------------------------------------GAATGGAAATTAAGTCCAATGAAAGATGCTG<92

14001>TTCTTTTGATGGAGATGCAGACAGAATTGTTTATTACTACCATGATGCAGATGGCCACTTTCATCTCATAGATGGAGACAAGATAGCAACGTTAATTAGC>14100
  510>TTCTTTTGATGGAGATGCAGACAGAATTGTTTATTACTACCATGATGCAGATGGCCACTTTCATCTCATAGATGGAGACAAGATAGCAACGTTAATTAGC>609
   91<TTCTTTTGATGGAGATGCAGACAGAATTGTTTATTACTACCATGATGCAGATGGCCACTTTCATCTCATAGATGGAGACAAGATAGCAACG~~~~~~~~~<1
```

B

Alignment of patient RNA species with wt sequence

```
WT cDNA sequence                CTGTTTAATGATGGGTCCAAGGGCAAACTCAATCATTTATGTGGAGCTGACTTTGTGAAAAGTCATCAGAAACCTCCA>900
Healthy control forward         ---------------------------------------------------------------------ACCTCCA>9
Patient small fragment forward  --------------------------------------------------------------------AACCTCCA>15
Healthy control reverse         --------------------GGCAAACTCAATCATTTATGTGGAGCTGACTTTGTGAAAAGTCATCAGAAACCTCCA<126
Patient small fragment reverse  ---------------------GCAAACTCAATCATTTATGTGGAGCTGACTTTGTGAAAAGTCATCAGAAACCTCCA<111
```

Exon 7/8 junction

```
 901>CAGGGAATGGAAATTAAGTCCAATGAAAGATGCTGTTCTTTTGATGGAGATGCAGACAGAATTGTTTATTACTACCATGATGCAGATGGCCACTTTCATC>1000
  10>CAGGGAATGGAAATTAAGTCCAATGAAAGATGCTGTTCTTTTGATGGAGATGCAGACAGAATTGTTTATTACTACCATGATGCAGATGGCCACTTTCATC>109
  16>CAGGGAATGGAAATTAAGTCCAATGAAAGATGCTGTTCTTTTGATGGAGATGCAGACAGAATTGTTTATTACTACCATGATGCAGATGGCCACTTTCATC>115
 125<CAGGGAATGGAAATTAAGTCCAATGAAAGATGCTGTTCTTTTGATGGAGATGCAGACAGAATTGTTTATTACTACCATGATGCAGATGGCCACTTTCATC<26
 110<CAGGGAATGGAAATTAAGTCCAATGAAAGATGCTGTTCTTTTGATGGAGATGCAGACAGAATTGTTTATTACTACCATGATGCAGATGGCCACTTTCATC<11

1001>TCATAGATGGAGACAAGATAGCAACGTTAATTAGCAGTTTCCTTAAAGAGCTCCTGGTGGAGATTGGAGAAAGTTTGAATATTGGTGTTGTACAAACTGC>1100
 110>TCATAGATGGAGACAAGATAGCAACGTTAATTAGCAGTTTCCTTAAAGAGCTCCTGGTGGA~~~~~~~~~~~~~~~~~~~~~~~~~~~~~~~~~~~~~~~>170
 116>TCATAGATGGAGACAAGATAGCAACGTTAATTAGCAGTTTCCTTAAAGAGCTCCTGGTGGA--------------------------------------->176
  25<TCATAGATGGAGACAAGATAGCAAC~~~~~~~~~~~~~~~~~~~~~~~~~~~~~~~~~~~~~~~~~~~~~~~~~~~~~~~~~~~~~~~~~~~~~~~~~~~<1
  10<TCATAGATG-------------------------------------------------------------------------------------------<2
```

Figure S2. Alignment of the sequence of PCR fragments from patient samples using a forward primer binding in exon 7 and the reverse primer binding in exon 8. A) The sequence of the large PCR fragment with partial intron

7 retention aligned to the genomic sequence. The A>G mutation in the intron sequence is indicated by the arrow. Only the relevant part of intron 7 is included

B) The sequence of the small PCR fragment corresponding to the wt mRNA sequence.

The exon 7/8 junction is indicated by the red arrow.

Figure S3

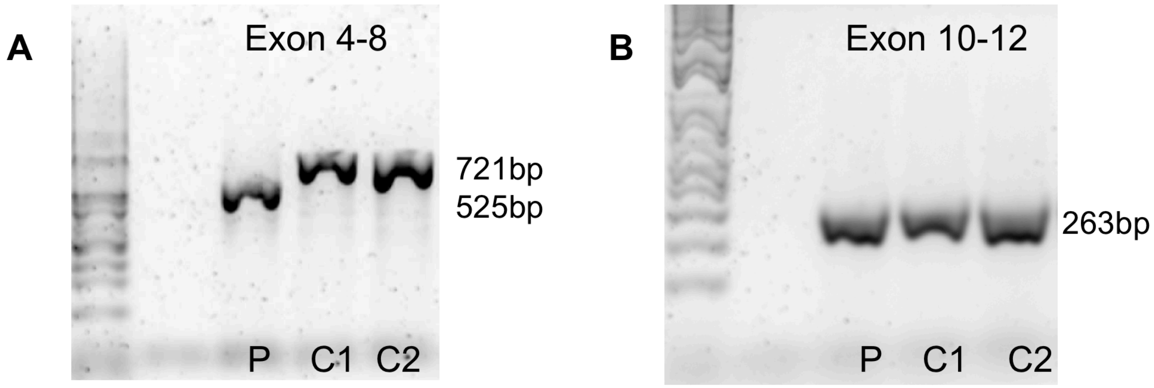

Figure S3. RT-PCR performed on mRNA from peripheral blood and EBV-transformed B-cells. Agarose gel analysis of RT-PCR performed on cDNA from patient cells (P) and from two healthy controls (C1 and C2). The PCRs were performed using A) forward primer in exon 4 and reverse primer in exon 8, B) forward primer in exon 10 and reverse primer in exon 12.

Figure S4

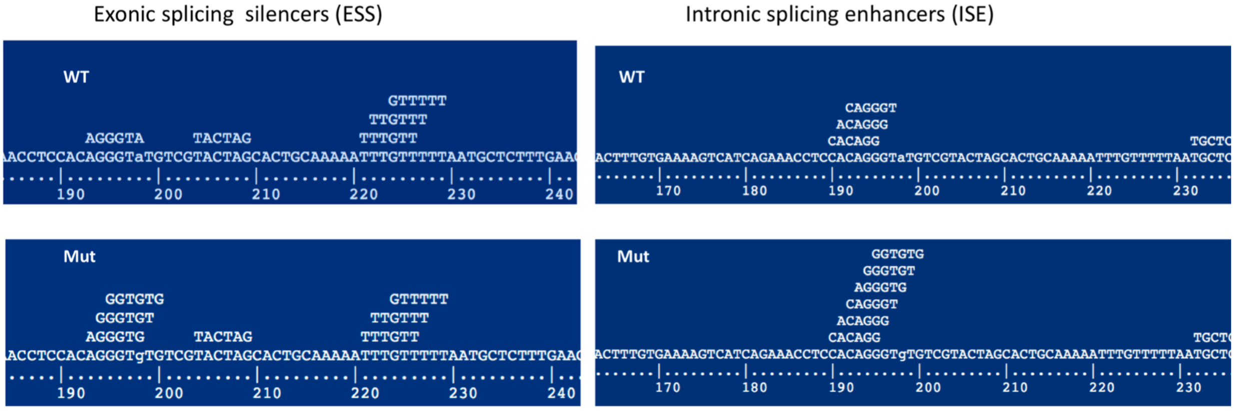

Figure S4. Identified exonic splicing silencers (ESS) and intronic splicing enhancers(ISE) around the mutation site. The wild type sequence is shown in the top panels and the patients sequence in the bottom panels. The splice site is in position196 (GG/GTaT) with the mutated base in lower case. The mutation increase the presence of ESS sites as identified using the on line toolACESCAN2, http://genes.mit.edu/cgi-bin/acescan2.

MATERIAL AND METHODS

Whole exome sequencing
Genomic DNA was prepared and quantified using Qubit 2.0 Fluorimeter (Invitrogen), fragmented into average 300-bp fragments using E220 focused-ultrasound sonicator (Covaris) and 3 μg of the fragmented DNA was converted into sequencing ready library using SureSelect XT library preparation and the Clinical Research Exome bait set (Agilent Technologies). The obtained library was quantified using Qubit 2.0 and sequenced on HiSeq 2500 (Illumina) in rapid-run mode. Paired end sequencing was carried out to yield 85 M read pairs corresponding to an average coverage of 250x.

Bioinformatic analysis of the sequence data was carried out using v1.5.6 of Mutation Identification Pipeline (MIP) [1]. MIP aligns the sequence reads, calls variants and annotates the variants with information from several databases. MIP then considers the annotated information and the genetic inheritance pattern when calculating a rank score for each variant. For clinical interpretation, the variants are sorted according to the rank score and assessed in decreasing order. The sequence reads were aligned to the whole human genome reference GRCh37 using Mosaic (v.2.2.3) and PCR and optical duplicates were marked using PicardTools (Picard 2013, http://picard.sourceforge.net:). GATK v.2.8-1 performed realignment, base recalibration, and variant identification [2]. The results were uploaded to a browser-based tool (Scout, in-house developed at Clinical Genomics facility at Science for Life Laboratory, Stockholm, Sweden) for clinical variant interpretation. The clinical interpretation was restricted to 305 genes associated to immunodeficiency and auto-inflammatory diseases (Supplementary Table S1).

Sanger sequencing
The following primers were designed to amplify a DNA sequence including the variant.
PGM3-Forward (Fw): CTGTCAGTTCAGCTGTTTAATG
PGM3-Reverse(Rv): AGCTGCAAGATTCTTAACTCAC.
PCR was performed in a volume of 20 μL including 20 ng of DNA, 200 nM of primer and 1x AmpliTaq Gold 360 Master Mix (Thermo Fisher Scientific). After an initial step of 95°C for 7 min, 34 cycles of PCR were conducted with denaturation at 94°C for 30 seconds (sec) followed by annealing at 60°C for 35 sec and elongation at 72°C for 45 sec The same primers were used for Sanger sequencing in both directions using the BigDye Terminator v3.1 sequencing kit (Thermo Fisher Scientific). The sequencing reaction was purified with the BigDye XTerminator Purification Kit (Thermo Fisher Scientific) according to the manufacturer's instructions, and run on the ABI 3130xl instrument. Results were analyzed using the Sequencing Analysis software (Thermo Fisher Scientific).

Establishing Epstein Barr Virus (EBV) transformed B-cells and in vitro cell culturing
Peripheral blood from the patient and from healthy controls (n=4) were collected in EDTA tubes, diluted 1:2 in RPMI1640 (Life Technologies), with 20 % DMSO, aliquoted in 1.2 ml/tube and frozen at -80 or -150°C until used for further analysis as previously described [3].

To establish EBV cell lines aliquots of frozen whole blood or cells from fresh blood were used. Frozen cells were thawed and washed in RPMI-20% FCS (RPMI-20) containing EDTA, while erythrocytes in freshly retrieved blood were lysed using IO-T3 lyse buffer (Beckman Coulter). Cells were then washed with RPMI-20 medium, counted and re-suspended in filtered EBV B95-8 supernatant (kindly provided by professor Ingemar Ernberg, Karolinska Institutet). Cells, 10^7/ml, were incubated with virus supernatant at 37° C for 90 min, pelleted, and re-suspended in RPMI-20, supplemented with 10 mM HEPES, 1x PEST (both from Life Technologies), and 1 μg cyclosporin A (Sigma) /ml, and divided in two wells in a U-bottomed 96-well plate. The plates were incubated under normal cell culture conditions (5% CO_2 and 95% humidified atmosphere) for several weeks. Half of the medium in the wells was exchanged every 4-5 days and after 2 weeks the cyclosporin was withdrawn. Control and patient samples were treated in parallel. No EBV transformed cells were recovered from frozen patient samples while easily recovered from all controls.

EBV transformation of fresh unfrozen cells from the patient blood was also tried and then the culture medium was conditioned by adding 50% sterile-filtered (0.2 μm) medium from established normal EBV cell cultures. Under these conditions EBV-transformed cells from the patient sample could eventually be established. Cells were expanded and maintained in RPMI-20 supplemented with 10 mM HEPES and 50 μg/ml Gentamicine (Life Technologies) to provide material for further analyzes.

Reverse Transcriptase (RT)-PCR and quantitative RT-PCR
RNA was extracted from peripheral whole blood and from EBV cells from patient and healthy controls using the Tri-reagent (Sigma) according to the manufacturer's protocol. cDNA was prepared using the High capacity cDNA

Reverse transcription (RT) Kit (Thermo Fisher Scientific) with random primers and 500 ng total RNA in each 20 µL reaction.

For PCR each reaction 1 µL cDNA and 1x AmpliTaq Gold 360 Master Mix were used and the same PCR conditions as described above. Primers binding in exons 4, 5, 7, and 10 combined with Rv primers in exons 4, 8, 10 and 12 were used as indicated in figures.
Quantitative RT-PCR was performed using the Power SYBR Green PCR master mix and a StepOne Plus real time PCR instrument (Applied Biosystems). A primer binding over the exon 7/8 junction (Fw e7/8) was used in combination with the Rv e8 primer to specifically amplify wild type (wt) mRNA. and related to the house keeping gene *HPRT* in order to calculate the relative amount of correctly spliced mRNA.

Primer sequences for RT-PCR:
Fw e 4   5'TGGTTGATCCTTTGGGTGA3'
Fw e 5 5'GATACCAGGCCCAGCAGTGA3'
Fw e 7 5'GCAAACTCAATCATTTATGTGG3';
FW e7/8 junction 5'CCTCCACAGGGAATGGAAA3'
Fw e 10 5'CACTAAGACTGGTGTAAAACATTTGC3';
Rv e4 5'TCACAGCTTCTTTCTCGCTGA3'
Rv e 8 5'TCCACCAGGAGCTCTTTAA3'
Rv e 10 5'GTTTTACACCAGTCTTAGTGCAA3'
Rv e 12 5'TCAAGCCCTTCAGAGCCAA3' .
Fw HPRT 5'GACTTTGCTTTCCTTGGTCAG
Rv HPRT 5'GGCTTATATCCAACACTTCGTGGG

Immunoblotting
Cells were lysed in RIPA buffer (50 mM Tris-HCl, pH 7.4 with 50 mM sodium fluoride, 150 mM sodium chloride, 2 mM EDTA, 1.0% NP-40, 0.5% sodium deoxycholate, and 0.1% sodium dodecyl sulfate (SDS)), supplemented with EDTA-free complete protease inhibitor cocktail (Roche). The lysates were separated on a SDS BIS-TRIS 4-12% polyacrylamide gels (Life Technologies) and blotted to cellulose filter using the iBlot dry blotting system (Life Technologies). Staining for specific proteins was made using rabbit anti-PGM3 (PA5-22353, Thermo Scientific) and mouse anti-actin (A-5441, Sigma) and with the secondary antibodies goat anti-mouse-800CW and goat anti-rabbit-680, respectively (both from LiCor Biosciences). The signals were quantified using the Odyssey imaging system (LiCor Biosciences) and the PGM3 relative to actin levels were calculated.

References
[1] H. Stranneheim, M. Engvall, K. Naess, N. Lesko, P. Larsson, M. Dahlberg, R. Andeer, A. Wredenberg, C. Freyer, M. Barbaro, H. Bruhn, T. Emahazion, M. Magnusson, R. Wibom, R.H. Zetterstrom, V. Wirta, U. von Dobeln, A. Wedell, Rapid pulsed whole genome sequencing for comprehensive acute diagnostics of inborn errors of metabolism, BMC Genomics, 15 (2014) 1090.
[2] A. McKenna, M. Hanna, E. Banks, A. Sivachenko, K. Cibulskis, A. Kernytsky, K. Garimella, D. Altshuler, S. Gabriel, M. Daly, M.A. DePristo, The Genome Analysis Toolkit: a MapReduce framework for analyzing next-generation DNA sequencing data, Genome Res, 20 (2010) 1297-1303.
[3] K.E. Lundin, A. Hamasy, P.H. Backe, L.N. Moens, E. Falk-Sorqvist, K.B. Elgstoen, L. Morkrid, M. Bjoras, C. Granert, A.C. Norlin, M. Nilsson, B. Christensson, S. Stenmark, C.I. Smith, Susceptibility to infections, without concomitant hyper-IgE, reported in 1976, is caused by hypomorphic mutation in the phosphoglucomutase 3 (PGM3) gene, Clin Immunol, 161 (2015) 366-72.
